# Potential for adaptation to climate change: family-level variation in fitness-related traits and their responses to heat waves in a snail population

**DOI:** 10.1186/s12862-017-0988-x

**Published:** 2017-06-15

**Authors:** Katja Leicht, Katri Seppälä, Otto Seppälä

**Affiliations:** 1 Eawag, Swiss Federal Institute of Aquatic Science and Technology, 8600 Duebendorf, Switzerland; 2 Department of Biological and Environmental Science, University of Jyvaskyla, 40014, Jyvaskyla, Finland; 3 ETH Zürich, Institute of Integrative Biology (IBZ), 8092 Zürich, Switzerland

**Keywords:** G × E interaction, Global warming, Great pond snail, Immunocompetence, Life history trait, Mollusc

## Abstract

**Background:**

On-going global climate change poses a serious threat for natural populations unless they are able to evolutionarily adapt to changing environmental conditions (e.g. increasing average temperatures, occurrence of extreme weather events). A prerequisite for evolutionary change is within-population heritable genetic variation in traits subject to selection. In relation to climate change, mainly phenological traits as well as heat and desiccation resistance have been examined for such variation. Therefore, it is important to investigate adaptive potential under climate change conditions across a broader range of traits. This is especially true for life-history traits and defences against natural enemies (e.g. parasites) since they influence organisms’ fitness both directly and through species interactions. We examined the adaptive potential of fitness-related traits and their responses to heat waves in a population of a freshwater snail, *Lymnaea stagnalis*. We estimated family-level variation and covariation in life history (size, reproduction) and constitutive immune defence traits [haemocyte concentration, phenoloxidase (PO)-like activity, antibacterial activity of haemolymph] in snails experimentally exposed to typical (15 °C) and heat wave (25 °C) temperatures. We also assessed variation in the reaction norms of these traits between the treatments.

**Results:**

We found that at the heat wave temperature, snails were larger and reproduced more, while their immune defence was reduced. Snails showed high family-level variation in all examined traits within both temperature treatments. The only negative genetic correlation (between reproduction and antibacterial activity) appeared at the high temperature. However, we found no family-level variation in the responses of most examined traits to the experimental heat wave (i.e. largely parallel reaction norms between the treatments). Only the reduction of PO-like activity when exposed to the high temperature showed family-level variation, suggesting that the cost of heat waves may be lower for some families and could evolve under selection.

**Conclusion:**

Our results suggest that there is genetic potential for adaptation within both thermal environments and that trait evolution may not be strongly affected by trade-offs between them. However, rare differences in thermal reaction norms across families indicate limited evolutionary potential in the responses of snails to changing temperatures during extreme weather events.

**Electronic supplementary material:**

The online version of this article (doi:10.1186/s12862-017-0988-x) contains supplementary material, which is available to authorized users.

## Background

Natural populations of numerous species are currently threatened by anthropogenic environmental changes such as habitat loss, chemical pollution, and invasive species. One of the factors with wide ecological effects is global climate change [1–4]. Climate change is a major threat for the Earth’s biodiversity, and the persistence of many populations will depend on their ability to respond to changing climatic conditions by means of (1) range shifts, (2) phenotypic plasticity, and/or (3) evolutionary adaptations. Of these, evolutionary adaptations are potentially of very high importance since climate change imposes intensified and novel selective pressures on organisms. This is true even in the case of range shifts and phenotypic plasticity because first, individuals that disperse into new areas are likely to experience altered selection owing to, for instance, different photoperiod (determined by latitude) and species interactions (all species are unlikely to disperse the same way) [5]. Second, phenotypic plasticity is unlikely to be optimal outside those environmental conditions under which it evolved [6] and it may be costly, with negative fitness effects [7, 8].

A necessary prerequisite for evolutionary change by means of natural selection is within-population heritable genetic variation in the traits subject to selection. Typically, fitness-related traits are considered to show heritable variation and thus evolutionary potential in nature [9]. This is also the case with some of the traits examined under climate-change mediated selection since phenological traits often show significant heritability (reviewed in [5]). Studies on key defence traits against altered climatic conditions (e.g. heat and desiccation resistance), however, indicate limited or even complete lack of genetic variation (e.g., [10–12], but see [13]). Furthermore, even if the traits under selection are heritable, the expression of genetic variation in them may be environment-dependent so that genetic variation found under benign environmental conditions is reduced in challenging environments [14]. Thus, if environmental change that leads to strong selection also reduces the expression of genetic variation, this will limit its expected response to selection [15]. Similarly, the strength and/or sign of genetic covariation among traits may change [16], which could also affect trait evolution under climate change conditions [17].

So far, few empirical studies have examined genetic variation in organisms’ performance in relation to changing climatic conditions (see references above), and they have focused on a limited set of traits (mainly phenology). Therefore, a need to investigate adaptability to climate change across a broader range of traits as well as organisms remains. It is especially important to consider evolutionary potential in life-history traits and defences against natural enemies (e.g. parasites) since they can strongly influence organisms’ fitness both directly and through species interactions. Here, we experimentally examined within-population genetic variation at family level in such traits at typical and heat wave temperatures as well as in their thermal reaction norms between the temperature treatments in a freshwater snail, *Lymnaea stagnalis*. We chose to focus on heat waves since their increasing frequency and severity has been suggested to have an even greater impact on natural populations than the gradual increase in average temperatures [18–20]. We quantified snail size, fecundity and immune function because of their high importance for fitness and their potential to alter species interactions (host–parasite relationships). Using multiple traits also allowed us to test environment-dependence of family-level covariation among them.

## Results

**Fig. 1:**
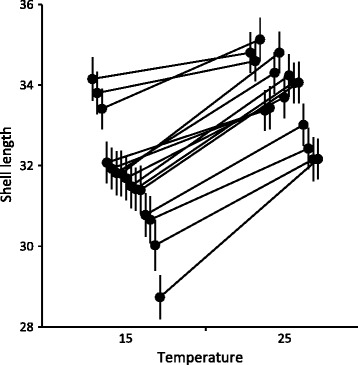
Shell length (mm) of *L. stagnalis* snails. Estimated marginal means (± SE) for 15 families after maintained in two temperature treatments (15 °C, 25 °C) for seven days. Families are arranged according to their rank order (from largest to smallest) at 15 °C, and they are connected between the treatments using reaction norms

**Fig. 2:**
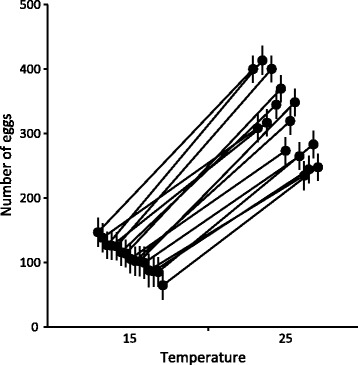
Number of eggs oviposited by *L. stagnalis* snails. Estimated marginal means (± SE) for 15 families after maintained in two temperature treatments (15 °C, 25 °C) for seven days. Families are arranged according to their rank order (from highest to lowest) at 15 °C, and they are connected between the treatments using reaction norms

Snails exposed to 25 °C were larger at the end of the study compared to snails maintained at 15 °C (Table 1, Fig. 1). They were also more likely to oviposit (97.6 ± 0.9% versus 85.8 ± 2.0% at 15 °C; GLM: d.f. = 1, Wald *χ* ^2^ = 20.807, *p* < 0.001) and they produced a higher number of eggs (Table 1, Fig. 2). Snail size and the number of oviposited eggs also showed significant family-level variation (Table 1, Figs. 1 & 2). Their responses to the high temperature were, however, consistent across families, indicated by statistically non-significant interaction terms between temperature and family (Table 1) and mainly parallel reaction norms between the temperature treatments (Figs. 1 & 2).

**Table 1:** ANOVAs for shell length, number of oviposited eggs, and immune parameters

	Source	d.f.	MS	*F*	ƞ^2^ (%)	*P*
shell length	temperature (T)	1	569.909	98.839^a^	14.2	<0.001
	family (F)	14	45.502	7.889^a^	15.9	<0.001
	block	5	33.439	6.608	4.2	<0.001
	T × F	14	5.769	1.140	2.0	0.319
	error	503	5.060			
number of oviposited eggs	temperature (T)	1	165.410	697.287^a^	53.4	<0.001
	family (F)	14	1.491	6.286^a^	6.7	0.001
	block	5	0.627	2.699	1.0	0.020
	T × F	14	0.237	1.021	1.1	0.431
	error	503	0.232			
haemocyte concentration	temperature (T)	1	1.500	2.357^a^	0.6	0.147
	family (F)	14	1.600	2.511^a^	9.5	0.048
	block	5	1.574	4.077	3.4	0.001
	T × F	14	0.638	1.652	3.8	0.062
	error	503	0.386			
PO-like activity	temperature (T)	1	2.013	9.270^a^	2.7	0.009
	family (F)	14	0.419	1.927^a^	7.8	0.116
	block	5	0.621	5.141	4.2	<0.001
	T × F	14	0.218	1.802	4.1	0.035
	error	503	0.121			
antibacterial activity	temperature (T)	1	4325.888	98.056^a^	11.8	<0.001
	family (F)	14	311.926	7.074^a^	11.9	<0.001
	block	5	243.372	4.700	3.3	<0.001
	T × F	14	44.081	0.851	1.7	0.613
	error	503	51.785			

^a^T × F as the error term

Factors are water temperature (15 °C, 25 °C), family (15 families), and block (6 blocks). The effect size ƞ^2^ shows the proportion of total variance explained by each factor

**Fig. 3:**
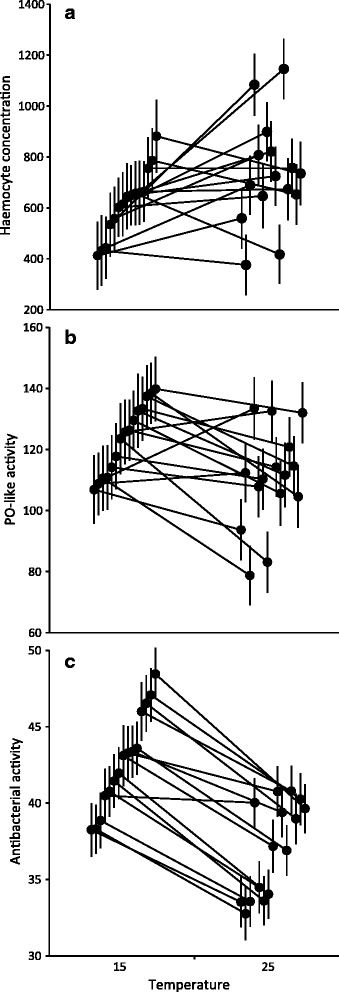
Immune activity of *L. stagnalis* snails. (**a**) Haemocyte concentration (cells/μl; estimated marginal means ± SE), (**b**) phenoloxidase (PO)-like activity (increase in optical density in miliunits; estimated marginal means ± SE), and (**c**) antibacterial activity (decrease in optical density in miliunits; estimated marginal means ± SE) in 15 families after maintained in two temperature treatments (15 °C, 25 °C) for seven days. Families are arranged according to their rank order (from lowest to highest) at 15 °C, and they are connected between the treatments using reaction norms

Exposure to 25 °C reduced snail immune defences (Table 1, Fig. 3). However, examined immune parameters differed in their responses to temperature (Table 1, Fig. 3). Haemocyte concentration of snail haemolymph did not differ between snails maintained at 25 °C and 15 °C, while PO-like activity and antibacterial activity were reduced at the high temperature (Table 1, Fig. 3). We found significant family-level variation in all immune traits (Table 1, Fig. 3; separate ANOVAs for PO-like activity at each temperature: 15 °C: *F*_14, 234_ = 1.743, *p* = 0.048; 25 °C: *F*_14, 264_ = 3.917, *p* < 0.001). Furthermore, family-level variation in PO-like activity interacted with temperature, indicated by a significant family-by-temperature (i.e. G × E) interaction (Table 1) and crossing reaction norms between the treatments (Fig. 3b). This suggests genetic variation in the response of snails to high temperatures in this trait.

At 15 °C, we found significant positive genetic correlations between snail shell length and PO-like activity as well as between shell length and the number of oviposited eggs (Table 2). At 25 °C, the relationship between shell length and PO-like activity was non-significant while the observed positive genetic correlation between shell length and the number of oviposited eggs remained (Table 2). Furthermore, haemocyte concentration and PO-like activity were positively, and the number of oviposited eggs and antibacterial activity were negatively correlated at 25 °C (Table 2). The observed family-level variation/covariation in the examined traits was unlikely to be due to differences in neutral genetic variation since snail families did not differ in heterozygosity, estimated using microsatellite markers (Additional file 1).

**Table 2:** Genetic correlations (± SE) between shell length, number of oviposited eggs, and immune parameters

	Shell length	Number of eggs	Haemocyte concentration	PO-like activity	Antibacterial activity
shell length		0.969*** (±0.017)	**0.175 (±0.444)**	0.708** (±0.192)	**−0.153 (±0.267)**
number of eggs	0.925*** (±0.048)		**−0.110 (±0.538)**	**0.470 (±0.356)**	**0.160 (±0.317)**
haemocyte concentration	0.140 (±0.315)	0.079 (±0.306)		**−0.039 (±0.734)**	**−0.362 (±0.455)**
PO-like activity	0.418 (±0.273)	0.329 (±0.283)	0.956*** (±0.026)		**−0.348 (±0.387)**
antibacterial activity	−0.453 (±0.273)	−0.641** (±0.194)	−0.079 (±0.317)	−0.270 (±0.304)	

Estimates are calculated separately at 15 °C (bold values above the diagonal) and 25 °C (values below the diagonal). Significance levels (*t*-tests): * *p* < 0.05, ** *p* < 0.01, *** *p* < 0.001

## Discussion

On-going global climate change poses a serious threat for natural populations unless they are able to evolutionarily adapt to changing environmental conditions [5, 21]. We examined family-level variation and covariation in life history and immune defence traits in a laboratory stock of a natural population of the freshwater snail *L. stagnalis* at typical (15 °C) and heat wave (25 °C) temperatures, as well as family-level variation in the responses of these traits to the high temperature (i.e. reaction norms between the treatments). Exposure to 25 °C increased size and reproductive output of snails while immune defence (PO-like and antibacterial activity, but not haemocyte concentration of haemolymph) was reduced. We found high family-level variation in all examined traits in both temperature treatments. The observed genetic correlations among traits were mainly positive. One negative genetic correlation (between reproduction and antibacterial activity of haemolymph) was found at 25 °C. These findings suggest that high temperatures may not strongly reduce the potential of the examined traits to respond to natural selection. However, the responses of most traits to the high temperature were similar across snail families, indicated by parallel reaction norms. Only the negative effect of the high temperature on one immune defence trait (PO-like activity) showed family-level variation, which indicates that for some families the cost of exposure to high temperatures is lower and hence could be selected for.

Temperature-induced phenotypic changes in fitness-related traits are common in nature (e.g., [22–24]). The observed increase in the reproduction and size of snails when exposed to elevated temperatures is also found in other ectotherms (e.g., [25–28]). These effects are most likely due to high metabolic activity, which can increase the amount of resources allocated to reproduction and growth [25]. The observed decrease in immune activity at the high temperature is also in line with the results from other taxa (e.g., [28–32]) and may be due to altered resource allocation towards growth and reproduction rather than immune defence (see [33]). Variation in the responses of different immune parameters to high temperatures (see also [24, 34]) could be a result of the other functions of some immune traits beside defence (e.g. involvement of haemocytes in nutrient transport and repair mechanisms), which could change the hierarchy of resource allocation among them (see [33]).

We found high family-level variation, suggesting genetic variation and thus adaptive potential in all examined traits in both temperature treatments. It is important to note that in studies utilising maternal sibships, such variation could arise not only due to additive genetic variance but also due to dominance variance and/or non-genetic maternal effects (see [35]). In our study system, however, the latter effects have been reported to be very weak in determining the variation in the examined traits [36]. In studies conducted using other organisms, the effect of challenging environmental conditions on the amount of genetic variation in key life history traits in natural populations has been shown to vary [14]. However, most studies have reported only limited genetic variation in growth and reproduction of organisms under harsh environmental conditions (see [37, 38]). This indicates that lack of adaptive potential could prevent evolutionary responses to climate-mediated selection in many species. For instance, in *Drosophila*, especially species with narrow geographical distribution show very little genetic variation in desiccation and cold resistance [10, 11]. In other taxa, also widely distributed species show genetic limitations for evolutionary responses [12]. Possible reasons for this are, among others, local adaptation, bottleneck events, or historical events of strong directional selection [39, 40].

In our study, genetic correlations between the examined traits indicated a genetic trade-off only between reproduction and antibacterial activity of haemolymph. Interestingly, this negative genetic correlation appeared only at the high temperature. The emergence of trade-offs when exposed to challenging conditions could be due to resources becoming scarce when protection and repair mechanisms are activated, which can lead to allocation trade-offs among traits [41, 42]. A potential evolutionary consequence of such environment-dependent trade-offs is limited responses of traits to selection under climate change [16, 43]. In this study, negative genetic correlations were scarce, and thus adaptation processes at high temperatures may not be significantly limited by trade-offs in this system. It is, however, possible that the examined traits are traded off with some other traits.

In spite of high family-level variation in the examined traits, genetic variation in their responses to the high temperature was limited (i.e. reaction norms between the temperatures were largely parallel). This is in line with studies on birds that found plastic responses, but little evidence for heritability of the slope of thermal reaction norms for reproductive traits such as laying date and clutch size, which indicates limited potential for temperature-mediated evolutionary responses in these traits (e.g., [44–46]). However, in other studies, genetic variation in thermal reaction norms for life history traits (e.g. growth rate, timing of reproduction) is found and is suggested to provide the genetic potential for adaptive responses to high temperatures [47–49]. We found family-level variation in the response of snails to a heat wave temperature only in one of the examined immune traits (PO-like activity). This suggests that the price snails need to pay for increased size and reproduction by compromising immune defence may be variable across individuals with different genetic backgrounds and thus show adaptive potential. Therefore, families experiencing the lowest costs may be selected for.

## Conclusions

*L. stagnalis* snails responded to an experimental heat wave by increasing reproduction and size while reducing expression of immune defence traits. Most of these traits showed high family-level variation at both control and heat wave temperatures. However, responses to the high temperature were similar in most traits across different families (i.e. parallel reaction norms). Only PO-like activity showed family-level variation in reaction norms between temperatures, which suggests that the cost snails need to pay for increased reproduction and size shows genetic variation and thus potential for adaptive evolution. The only genetic trade-off was found at the high temperature. Thus, our results suggest abundant adaptive potential in the examined traits, but only limited genetic variation in their thermal reaction norms.

## Methods

### Experimental animals

The hermaphroditic snail *L. stagnalis* inhabits lakes and ponds throughout the Holarctic region. When exposed to high temperatures it is known to initially increase reproduction and growth [33]. After 1 week, however, high reproductive rate ceases and the immune function weakens [33, 50]. This indicates that snails are not able to maintain increased performance when exposed to high temperatures over long time periods. *L. stagnalis* is an important host for various parasites, including several highly virulent trematode species [51, 52] that castrate the snails and increase their mortality [53, 54]. Thus, the reduced immune defence of snails at high temperatures [33, 50] can have wide implications for natural snail populations.

The snails we used for breeding experimental snails came from a laboratory stock population (F_3_ generation) originating from a pond in Zürich, Switzerland (47°22′ N, 8°34′ E). In this region, the summer water temperature in ponds typically remains low (< 16 °C), although it depends on the hydrology of the ponds (T. Salo, 2015, unpublished data). During heat waves, water temperature can, however, rapidly increase to 20–30 °C and remain high for over 2 weeks (T. Salo, 2015, unpublished data). To start the stock population, we collected 45 adult snails from the pond. Since *L. stagnalis* prefers outcrossing [55, 56], may engage in multiple matings [56], and can store sperm from those matings for over 2 months [57], the stock population can be assumed to reflect the genetic variation in the source population well. We maintained the stock population at 15 ± 2 °C (control temperature used in the experiment; see below) and kept its size at approximately 400 individuals to avoid loss of genetic polymorphism due to genetic drift (see [58]).

We haphazardly collected 15 adult snails (shell length > 20 mm) from the stock population to produce experimental snails. We maintained these snails individually in 0.2 L perforated plastic cups that were sunk into a water bath (15 ± 1 °C) connected to a biological filter. We fed the snails with fresh lettuce ad libitum and maintained them until each of them had produced two egg clutches (> 30 eggs per clutch). We used these clutches to obtain family lines (maternal sibships) for the experiment and transferred each clutch individually into plastic containers with 6 L of aged tap water (15 ± 1 °C). After the juveniles hatched, we fed them with *Spirulina* until they reached approximately 5 mm shell length. After that, we fed the snails with spinach and lettuce ad libitum*.* We changed water in the containers once a week. When the snails from all families were mature (shell length > 20 mm), we randomly chose 40 snails per family and placed them individually in 0.2 L perforated plastic cups sunk into a water bath (15 ± 1 °C) as above. Since pathogen resistance in snails may show age-related patterns [59], it is important to note that the difference in age of the experimental snails was not more than a week. We fed the snails with fresh lettuce ad libitum for 5 days before the experiment (see below) to acclimatize them to the experimental conditions. Since *L. stagnalis* is a simultaneous hermaphrodite [60] and can store allosperm for several weeks [57], snails did not need a mating partner to reproduce under these conditions.

Using maternal sibships, our goal was to estimate whether the responses of snails to high temperatures vary across different genetic backgrounds (i.e. families), and how much of the phenotypic variation in the examined traits is expressed at the family level. It is important to note, however, that variation among maternal sibships may not directly reflect the amount of additive genetic variation in the examined traits. This is because variation among families could also be due to other genetic factors such as dominance variance, and/or due to non-genetic maternal effects (reviewed in [35]). It is also possible that some families may have been multiply sired (see [56]). In our study system, however, phenotypic variation in immune function across full-sib families is known to reflect variation in the genetic background of snails rather than variation in non-genetic maternal effects [36]. Furthermore, the examined immune traits do not respond to inbreeding, which indicates a lack of directional dominance [36]. If multiple paternities within family lines were common, then the chances of finding differences among families would be reduced because among-family variation would be confounded by increased within-family variation.

### Experimental design

We maintained the snails as described above and randomly exposed them to two different temperature treatments (15 ± 1 °C and 25 ± 1 °C). We chose 25 °C as a high (i.e. heat wave) temperature as it lies above the thermal optimum of snails [61] and occurs intermittently in ponds inhabited by snails during hot summers (see above). We used 15 °C as a control temperature as it is close to the thermal optimum of snails [61] and common in ponds (see above). At the beginning of the experiment, we transferred the snails to their treatment temperatures in cups filled with aged tap water at 15 °C. This allowed a slow change to the target temperature for snails exposed to the high temperature treatment (over 10 h). We then exposed snails to their respective temperature treatments for 7 days. We chose a one-week exposure time as it represents a typical heat wave in Western Europe (average: 8.4 days [19]) and because the negative effects of high temperature on snails appear at that point [33]. After that we measured the shell length (to the nearest 0.1 mm), reproduction and immune function (described below) of each snail.

To examine whether possible family-level variation in traits studied could be due to differences in neutral genetic variation caused, for example, by inbreeding, we assessed heterozygosity at microsatellite loci in snail families, as described in Additional file 1. We also followed the survival of the snails daily throughout the experiment. Since the mortality of snails during the experiment was generally low [three snails (0.5% of all individuals) died during the experiment], survival could not be used as a response variable to examine the effects of experimental treatments. Additionally, not all traits could be measured from 23 snails (3.8% of all individuals) because of human errors during the measurements. We excluded these snails from the data. We conducted the experiment in six blocks, each of which consisted of three to four snails per family by treatment combination. We started the blocks on six consecutive days.

### Reproduction

To estimate the reproductive output of snails, we collected all the egg clutches oviposited by the snails during the experiment and photographed them from approximately 10 cm above with a Fujifilm FinePix F30 digital camera (scene mode: close up, focal length: 35 mm, aperture: F/2.8, shutter speed: 1/85, sensitivity: ISO-200, image size: 2848 × 2136 pixels, focus mode: auto focus). From each picture, we measured the two-dimensional area of the clutch that contained eggs using ImageJ software (ImageJ 1.42q, Wayne Rasband, National Institute of Health, USA). Then, we measured the area containing 10 eggs in each clutch and calculated an estimate of the total number of eggs in the clutch. We summed up the number of eggs in the clutches oviposited by each snail to get a measure of its reproductive output during the experiment.

### Immunological measurements

We measured haemocyte concentration, PO-like activity, and antibacterial activity of snail haemolymph to examine variation in snail immune function. In invertebrates, haemocytes, through phagocytosis, constitute the main part of the cellular immune response [62]. Furthermore, haemocytes can synthesize pro-phenoloxidase (pro-PO), which in the active form PO catalyses oxidative defence against parasites [63]. Antibacterial enzymes as a further component of the innate immune defence are used against microorganisms [64]. We chose to measure snail immune function rather than their susceptibility to any specific parasite species because examining several immune parameters gives a broad estimate of host defences, whereas studies focusing on a certain host-parasite interaction are necessarily specific to the particular parasite species being used. Such studies can also be confounded by the direct effects of temperature on parasite transmission stages [65–68]. The examined immune parameters are central in the immune system of invertebrates, including molluscs [69–72] and are known to respond to various immune elicitors [73] as well as to be subject to natural selection [74] in *L. stagnalis*.

We took the haemolymph samples and measured the immune parameters as described in [33]. Briefly, we collected haemolymph by gently tapping the underside of the snail’s foot until it retreated into its shell, simultaneously releasing haemolymph through hemal pore [75]. We counted the haemocyte concentration of haemolymph (cells per μl) using a Neubauer haemocytometer (Blau Brand, Wertheim, Germany), and measured its PO-like and antibacterial activity spectrophotometrically using a microtiter plate reader (Infinite 200, Tecan, Salzburg, Austria). For the measurements of PO-like activity, we mixed haemolymph samples with L-Dopa, and measured the increase in optical density (OD) of the solution followed by an enzymatic reaction in which PO oxidizes L-Dopa to dopachrom. For the measurements of antibacterial activity, we mixed haemolymph samples with lyophilized *Escherichia coli* cells, and measured the decrease in OD of the solution followed by a reaction in which antibacterial enzymes lyse *E. coli* cells.

We measured all immune parameters twice from a subsample of snails (haemocyte concentration: *N* = 76; PO-like activity: *N* = 67; antibacterial activity: *N* = 64) to estimate repeatability (*R*) of the measurements. Repeatability describes the proportion of variance in a character occurring among, rather than within, individuals. We calculated it from variance components derived from an analysis of variance (ANOVA) where snail individual was used as a factor [76]. Repeatability of all parameters was high (haemocyte concentration: *R* = 0.985, *F*_76, 77_ = 33.630, *P* < 0.001; PO-like activity: *R* = 0.985, *F*_67, 68_ = 33.001, *P* < 0.001; antibacterial activity: *R* = 0.880, *F*_64, 65_ = 3.487, *P* < 0.001).

### Statistical analyses

We performed all statistical analyses using IBM SPSS 23.0 (IBM, Armonk, NY, USA) software. To examine the effect of temperature on the probability of snails reproducing during the experiment, we used a generalized linear model (GLM) with the reproductive status of snails (oviposited, did not oviposit) as a binomial response variable. In the analysis, we used temperature as a fixed factor. Since there was no variation in some families and blocks (i.e. all individuals reproduced) we could not include family and block as factors in the model. After that, we analysed the variation in the number of oviposited eggs using only those snails that reproduced using an ANOVA. In the model, we used temperature as a fixed and family as a random factor. Additionally, we included the main effect of block (random factor) to reduce possible noise in the data that could arise from examining the snails on different days. To meet the assumptions of ANOVA, we ln transformed the number of produced eggs.

To analyse the variation in snail size and immune parameters (haemocyte concentration, PO-like activity, antibacterial activity of haemolymph) we used similar ANOVAs as above. To meet the assumptions of ANOVA, we ln transformed haemocyte concentration and PO-like activity. When statistically significant temperature-by-family interactions were observed in the above analyses, we analysed the variation in response variables separately for different temperatures. We did this to examine whether family-level variation was observed under both environmental conditions. Snails that did not reproduce were excluded from the analyses of snail size and immune parameters. This was done because reproduction can be traded off with these traits [77] and the decision not to oviposit may strongly alter their expression compared to snails that did oviposit.

In addition to the above ANOVAs, we examined the variation in responses of snails to temperature treatments by analysing the data using the approach in [78]. We formed random pairs of snails between temperature treatments within each family by block combination, and subtracted the trait values (egg number was standardized as described in [78]) of the snail maintained at 15 °C from those of the snail maintained at 25 °C in each pair. We then analysed the variation in calculated differences in each trait using ANOVAs with family and block as random factors. As this approach gave qualitatively similar results compared to the G × E interactions in the above ANOVAs, these results are not presented.

We calculated genetic correlations among traits separately in both temperature treatments using components of covariance and variance estimated, based on sums of cross-products and sums of squares in the analyses of covariance and variance, respectively [9]. We included the main effect of family and block as factors in all models. We calculated standard errors of estimated genetic correlations according to [43] as well as *t*-values to assess their statistical significance as the quotient of each genetic correlation and its standard error.

## Additional file

Additional file 1: Methods and results of the genetic analyses. (DOC 47 kb)
