# Supplementary material for: Potential for adaptation to climate change: family-level variation in fitness-related traits and their responses to heat waves in a snail population
Source: BMC Evol Biol. 2017 Jun 15;17:140. doi: 10.1186/s12862-017-0988-x (PMC5472919; doi:10.1186/s12862-017-0988-x)
Supplement: Additional file 1: — Methods and results of the genetic analyses. (DOC 47 kb) [file 12862_2017_988_MOESM1_ESM.doc]

**Additional file 1**

GENETIC ANALYSES

The level of heterozygosity estimated, for example, using neutral genetic markers often correlates positively with the fitness of organisms (reviewed in ). We estimated the heterozygosity of experimental snails using eight microsatellite loci (GenBank Accession No. AY225957, AY225958, AY225962, AY225963, EF208747 - EF208749, and EF208752 ). We chose six families for the analysis based on their overall performance so that three families with high trait values and three families with low trait values were used. From each family, we took tissue samples from the foot of the snails from 13 to 16 individuals. We extracted genomic DNA using Chelex® 100 resin (Sigma-Aldrich, St. Louis, MO, USA) . We amplified microsatellites in total volume of 15 μl per reaction in three separate multiplex reactions. Each reaction included 7.5 μl of Multiplex PCR Master Mix (Qiagen, Hilden, Germany), 6.6-7.1 μl RNase-free water, and 2 μl of extracted DNA. In multiplex reaction 1, we added 0.2 μM (F + R) of primer 2k33 and 0.4 μM of primer D5. In multiplex reaction 2, we added 0.2 μM (F + R) of primer 2k42, 0.4 μM of primer A112, and 0.6 μM of primer A102. In multiplex reaction 3, we added 0.4 μM (F + R) of primer 2k27 and C4, and 0.6 μM of primer 2k11. We carried out PCR reactions on a TProfessional Thermocycler (Biometra, Göttingen, Germany) using the following temperature cycling profile: a 15 min initial denaturing step at 95°C followed by 15 cycles with 30 sec at 94°C, 90 sec at 55°C, and 60 sec at 72°C, followed by 20 cycles with 30 sec at 94°C, 90 sec at 52°C, and 60 sec at 72°C, and a final extension step at 60°C for 30 min. Each PCR product (0.5 μl, 1:10 dilution) was mixed with 9.3 μl HiDi^TM^ formamide (Applied Biosystems, Woolston, Warrington, UK) and 0.2 μl GeneScan^TM^-500 LIZ size standard (Applied Biosystems, Woolston, Warrington, UK), and analysed on an ABI Prism 3130xl Genetic Analyzer (Applied Biosystems, Woolston, Warrington, UK). Alleles were identified using the software GeneMarker 1.8 (SoftGenetics, State College, PA, USA).

To estimate individual heterozygosity, we calculated the proportion of heterozygote loci (*x*/8) for each snail . After that, we calculated the mean heterozygosity for each family and analysed the variation in the heterozygosity using a generalized linear model (GLM). In the analysis, we used the proportion of heterozygote loci as a binomial response variable (logit link function) and family as a fixed factor.

RESULTS OF GENETIC ANALYSES

The mean heterozygosity of examined families lay between 0.56 and 0.73. The difference in heterozygosity among families was not statistically significant (GLM: Wald χ2 = 8.856, *P* = 0.115).

REFERENCES

1. Hansson B, Westerberg L. On the correlation between heterozygosity and fitness in natural populations. Mol Ecol. 2002;11:2467-74.

2. Knott KE, Puurtinen M, Kaitala V. Primers for nine microsatellite loci in the hermaphroditic snail *Lymnaea stagnalis*. Mol Ecol Notes. 2003;3:333-5.

3. Walsh PS, Metzger DA, Higuchi R. Chelex 100 as a medium for simple extraction of DNA for PCR-based typing from forensic material. BioTechniques. 1991;10:506-13.

4. Coltman DW, Pilkington JG, Smith A, Pemberton JM. Parasite-mediated selection against inbred Soay sheep in a free-living, island population. Evolution. 1999;53:1259-67.
